# High Prevalence of Nickel Allergy in an Overweight Female Population: A Pilot Observational Analysis

**DOI:** 10.1371/journal.pone.0123265

**Published:** 2015-03-30

**Authors:** Elena Angela Lusi, Vincenzo Maria Di Ciommo, Tommaso Patrissi, Paolo Guarascio

**Affiliations:** 1 St Vincent Private Hospital, Dublin, Ireland; 2 Ospedale Pediatrico Bambino Gesu’, Epidemiology and Biostatistics Unit, Rome, Italy; 3 Central Laboratory, Cardiology and Preventive Medicine Unit, Italian Red Cross, Rome, Italy; 4 Liver Unit, St Camillo-Forlanini Hospital, Rome, Italy; University of Chieti, ITALY

## Abstract

**Context:**

In our Allergy Unit, we incidentally observed that a low Nickel diet, prescribed for delayed allergy to Nickel sulfate, reduced body mass index (BMI) and waist circumference in overweight patients.

**Objectives:**

This pilot cross-sectional analysis was undertaken to compare the prevalence of Nickel allergy of overweight individuals versus the general population. We also had the chance to report the efficacy of a low Nickel diet on BMI and waist circumference in Nickel-sensitive overweight subjects.

**Methods:**

Eighty-seven overweight subjects, with a BMI >26 Kg/m^2^, were consecutively enrolled in a health prevention program, and screened for the presence of Nickel allergy. The enrolled population was mostly females (72/87) (82.8%). Forty-three overweight women and two men showed a Nickel allergy and started a low Nickel diet. After 6-months of dieting, 24 overweight allergic women could be traced and changes in BMI and waist circumference were calculated.

**Main Outcome Measurements:**

Prevalence of Nickel allergy in overweight.

**Results:**

Prevalence of Nickel allergy in overweight female was 59.7%, compared with a prevalence rate of 12.5% in the general population. A significant reduction in BMI was observed in 24 out of 43 overweight females with Nickel allergy after 24 weeks of a low Nickel diet. Relative to baseline, mean BMI decrease was 4.2±0.5 (P <0.001) and the mean decline in waist circumference was 11.7±0.6 cm (P< 0.001).

**Conclusions:**

This pilot observational analysis showed a substantially higher prevalence of Nickel allergy among overweight females, especially those with metabolic syndrome and fatty liver disease. A normocaloric low Nickel diet was effective in reducing BMI in this population. Further research is strongly needed to confirm these preliminary findings.

## Introduction

Nickel is the fourth most used metal in the world and its consumption is forecast to grow being used for several industrial products and medical appliances (Roskill analysis). As a natural element of the earth's crust small amounts are found in water, soil, and natural foods. Major dietary source of Nickel is plant food. Plant tissues contain more Nickel than animal tissues [1–2]. Nickel is an ideal sensitizing agent and it is responsible for the highest incidence of skin sensitization in the industrialized world, even in the pediatric age [3–5]. However, Nickel allergy not only affects the skin, but also results in systemic manifestations. Systemic Nickel allergy syndrome, is clinically characterized by cutaneous and systemic symptoms (such as headache, asthenia, itching, and gastrointestinal disorders related to histopathologic alterations of gastrointestinal mucosa) [6–8].

It has been estimated that the prevalence of Nickel allergy in the general population, is 8–15% for females and 1–3% for males [9–11]. However, in our Allergy Unit, we incidentally noticed an unusually high prevalence of Nickel allergy in overweight subjects, especially women with metabolic syndrome in the perimenopause age.

Therefore, we decided to undertake this pilot cross-sectional analysis to compare the prevalence of Nickel allergy of overweight individuals versus the general population. We also had the chance to report the efficacy of a low Nickel diet on BMI and waist circumference in Nickel-sensitive overweight subjects.

To our knowledge, this study is the first to evaluate the prevalence of Nickel allergy in overweight subjects and to evaluate the effectiveness of a low Nickel diet in reducing BMI.

## Materials and Methods

This study was carried out in the Allergy and Clinical Immunology Unit of the Central Laboratory of the Italian Red Cross, in Rome.

We enrolled 87 consecutive subjects (15 males, 72 females) with high BMI (>26 Kg/m^2^) referred to the Preventive Health Service of the Italian Red Cross for general health check-up and we screened this cohort for the presence of Nickel allergy. Anthropometric measurements (height, weight, BMI, waist circumference), blood pressure and biochemical variables (liver panels, fasting glucose, insulin, total cholesterol, triglycerides) were measured in each enrolled subject. Metabolic syndrome was diagnosed according to the modified NCEP ATP III criteria [12]. The presence of echogenic liver steatosis was also determined. Insulin resistance was estimated with the homeostasis model assessment index (HOMA), calculated as fasting glucose (mmol/L) times fasting insulin (mIU/L) divided by 22.5. Percentage of body fat was calculated as described previously [13]. Nickel allergy was diagnosed with a patch test performed with Nickel sulfate 5% (Lofarma Diagnostic) on scan pore disks, with delayed reactions evaluated 72 hours from disk application.

According to the current guidance for Nickel allergy treatment, a normocaloric balanced diet, formulated to be low only in Nickel (80–100 µg/daily), was prescribed to allergic overweight patients. The diet plan was developed by selecting low Nickel content foods from published database and following Sharma’s points as a guide [1]. We included traditional foods common in western diets and acceptable to the study population. Milk, eggs, all types of meat, fish, refined flower, vegetables and fruit with low nickel content were permitted. Instead legumes, soy, whole-grain products were excluded and the consumption of tomatoes and some vegetables (cauliflower, carrots, onions, spinach, lettuce) restricted. An allergist and a nearby nutritionist gave both verbal and written instruction of the diet.

Forty-three overweight allergic women and two allergic men started the diet. They had regulars appointments at 4, 8, 12 weeks. A follow-up of 24 weeks was established as final end point, to evaluate the long term efficacy of a low Nickel diet in reducing anthropometric indices.

## Statistical Analysis

### Prevalence study

In order to determine if the prevalence of Nickel allergy in our sample study group was higher than the prevalence observed in the general population, we used as test method the one-sample z test for proportions (Minitab Statistical Software).

### Analysis of BMI and waist circumference at the baseline and at six months

All data were analyzed using two-tailed paired sample *t* tests. A *P* value of less than 0.05 was considered significant.

## Ethics Statement

A specific ethical committee approval was not looked for because a Nickel-free diet is the current preventive precaution for patients with nickel allergy and implies very low health risk. Follow-up was entirely free from charge as other preventive advices from the Red Cross. Patients did not receive any payment for participation. Approval for this study was obtained from the IRB of the Italian Red Cross (N 0053214). Each subject’s consent was documented in the medical notes.

## Results

### Nickel Prevalence in Overweight Subjects

Demographic characteristics, anthropometric measurements and laboratory values of the enrolled population are described in Tables 1 and 2 respectively.

**Table 1:** Demographic characteristics of enrolled subjects at baseline.

Variables	TOTAL	Females	Males
**No. Overweight**	**87**	**72**	**15**
**Subjects with Nickel Allergy in the enrolled population**	**45**	**43**	**2**
**Subjects with Metabolic Syndrome**	**59**	**44**	**15**
**Subjects with Liver Steatosis**	**65**	**50**	**15**
**Subjects with Metabolic Syndrome + Liver Steatosis**	**51**	**36**	**15**
**Subjects with Nickel Allergy and Metabolic Syndrome**	**27**	**25**	**2**
**Subjects with Nickel Allergy and Liver Steatosis**	**31**	**29**	**2**
**Subjects with Nickel Allergy, Metabolic Syndrome + Liver Steatosis**	**24**	**22**	**2**

**Table 2:** Anthropometric measurements and laboratory values of enrolled subjects.

Variables	Mean(SD)
**Age (years)**	**54.2(11.9)**
**BMI**	**32.0(4.0)**
**Waist Circ. (cm)**	**104.5(10.8)**
**Tot Cholesterol (mg/dl)**	**226.7(45.2)**
**HDL (mg/dl)**	**51.6 (15.0)**
**Triglycerides (mg/dl)**	**152.3 (79.4)**
**Fasting Glucose (mg/dl)**	**110.4(35.9)**
**Fasting Insulin (μIU/ml)**	**14.0(11.8)**
**Weight(Kg)**	**86.5(15.9)**
**Height (cm)**	**163.5 (8.7)**
**HOMA-IR unit**	**2.4** * **7.5** °
**Body Fat %**	**43.2(6.7)**

*Median

**°Interquartile Interval**

**Fig 1:**
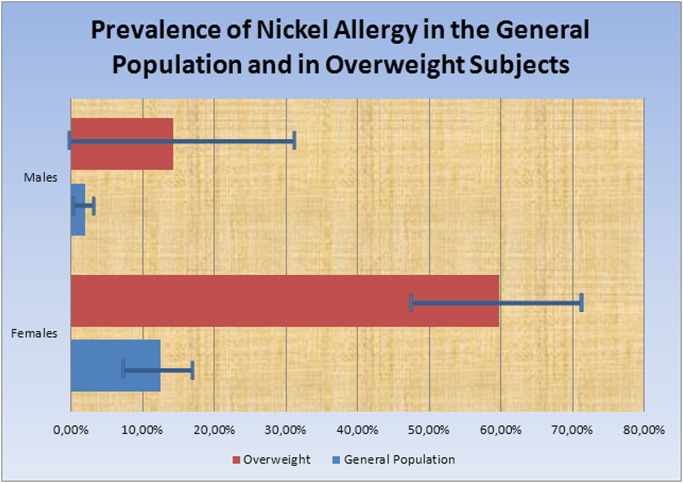
Prevalence of Nickel Allergy in overweight subjects (BMI≥26 kg/m^2^) compared to the general population [9]-[11].

The patch test showed a considerable higher prevalence of Nickel allergy in overweight patients compared to the general population. In particular, the prevalence of Nickel allergy among overweight women and males was 59.7% (43/72) (95% CI: 47.5% -71.1%; P<0.001) and 13.3% (2/15) (95% CI: 0–31.0%; P<0.1) respectively, in contrast to a prevalence rate of Nickel allergy of 8–15% in women and 1–3% in males in the general population [9–11] (Table 3), (Fig 1).

**Table 3:** Nickel Allergy Prevalence in our study sample of overweight subjects is significantly higher than in the general population.

Variables		Percentage	95% C.I.	P-value
**Nickel allergy in females**	**Our study population**	**59.7% (43/72)**	**47.5% -71.1%**	**<0.001**
	**General population**	**12.5%**		
**Nickel allergy in males**	**Our study population**	**13.3% (2/15)**	**0%– 31.0%**	**<0.1**
	**General population**	**2.0%**		

In the cases of overweight associated with metabolic syndrome and complicated by liver steatosis, the prevalence of Nickel allergy for females was slightly higher: 61.1% (22/36) (95% CI: 43%-77%).

### Effects of a Low Dietary Nickel on Anthropometric Indices at six months

Since males were too few, our statistical analysis included only the females population. At 24 weeks, of the 43 allergic overweight females, who started the low Nickel treatment, 24 were still following the diet and completed the follow-up.

Relative to baseline, mean BMI decrease was 4.2±0.5 (P <0.001) and the mean decline in waist circumference was 11.7±0.6 cm (P< 0.001) (Table 4).

**Table 4:** Changes in BMI, Body Fat and Waist circumference in 24 overweight females with Nickel allergy who completed the follow up at 6 months.

Variables	No. Females	Mean	S.D.	Standard Error	Mean difference	S.D. of the difference	Standard Error of the difference	95% C.I. for mean difference	P-value
**BMI at baseline**	**24**	**31.6**	**4.7**	**1.1**	**4.2**	**2.3**	**0.5**	**3.25%- 5.15%**	**<0.001**
**BMI at six months**		**27.4**	**3.5**	**0.9**					
**BODY FAT at baseline**	**24**	**44.8**	**6.7**	**1.5**	**5.1**	**2.8**	**0.6**	**4.03%- 6.17%**	**<0.001**
**BODY FAT at six months**		**39.6**	**5.8**	**1.4**					
**WAIST at baseline**	**24**	**113.8**	**10.1**	**4.1**	**11.7**	**1.5**	**0.6**	**10.5%- 12.9%**	**<0.001**
**WAIST at six months**		**102.2**	**10.2**	**4.2**					

The 19 overweight females were simply lost to follow-up. The main reasons for dropping out of the study were conflict with work schedule (15%) and having other health problems (13%). The rest of the dropouts were unavailable despite several phone calls. Interestingly, no subject complained about the diet and cited it as the reason for dropping out.

## Discussion

In our Allergy Unit, we realized that the majority of overweight people, especially women of menopausal age, had a Nickel allergy. In particular, overweight females showed a prevalence of Nickel allergy of 59.7%. If we consider overweight cases associated with metabolic syndrome and complicated by liver steatosis, the prevalence of Nickel allergy is slightly higher (61.1%). This is noteworthy, if we consider that the mean prevalence of Nickel allergy for females in the general population is 12.5% [9–11].

When a normocaloric diet, formulated to be low only in Nickel (100 µg/daily), was prescribed to allergic overweight females as the established therapy for their allergy, they manifested a reduction in body mass index and waist circumference in the first three months of diet, and this reduction was maintained stable at six months of follow up.

Is there a chance that Nickel could be related to overweight? Dipping back to the Seventies, biochemists already taught us that exposure of Nickel in animal models induces insulin overload, glycogenolysis and hyperglycemia in a concentration dependent manner [14–23]. Dose-dependent relationships between the amount of ingested Nickel and dermatitis with systemic symptoms has been demonstrated in various studies in humans [24]. Individual diets may vary, leading to substantial variability in Nickel intake. For example, if an individual consumed the following daily diet: breakfast—a bowl of oatmeal (0.22mg of Nickel) and one banana (0.04mg); lunch—one whole grain bun (0.01mg) with a chicken breast (0.01mg), green beans (0.03mg), and a Hershey’s bar (0.01mg); dinner—a serving of broccoli (0.02mg), a baked potato (0.02mg), and a pork chop (0.005mg); snack—a half-cup of peanuts (0.22mg); his or her Nickel intake for the day would be 0.58mg, which is well above the amount shown to cause flares [25–26].

A low Nickel diet is the standard therapy for sensitive patients with dermatitis associated with systemic manifestations.

However, the efficacy of a low Nickel diet in reducing BMI and waist circumference has never been described. Which could be the link between Nickel allergy, overweight and females in perimenopause?

A review of current research illustrates that overweight is a chronic inflammatory state and pro-inflammatory cytokine IL-17 is up regulated in the blood of obese humans [27–28]. IL-17 is also considerably produced by Nickel-specific T lymphocytes [29–30]. During menopause, estrogen deficiency induces the differentiation of IL-17 secreting Th cells [31–32]. Since Nickel is also a potent inducer of IL-17 production, it could be argued that an high dietary Nickel load may act in a synergistic way to increase the level of inflammation in allergic women during menopause. We can speculate that a young woman with a Nickel allergy is likely to develop a skin eczema and irritable bowel abnormalities, while a woman with a Nickel allergy in her menopause age is mostly like to gain weight and develop metabolic abnormalities.

Obesity is not only an immunological inflammation, but other factors appear to be important. Recent evidences suggest that the gut microbes play a role in determining the obese phenotype. Germ free mice are protected against obesity and the transfer of gut microbes from obese animals results in dramatic increase in body fat and insulin resistance in the recipients [33]. Many bacterial strains use Nickel in their growing process. Helicobacter pylori stores Nickel to aid its host colonization and a low Nickel diet enhances its eradication [34–35]. Is it conceivable that the excess of Nickel intake, not being essential in the body, is needed for the development of the gut’s microflora? An interesting hypothesis is that an high Nickel concentration select Nickel specific bacteria which in turn trigger an inflammation process in the gut of obese people. It therefore appears that Nickel may have pleiotropic roles in determining overweight: immunological, biochemical and microbiological.

The results of this pilot analysis are definitively preliminary and must be interpreted with caution, since the evaluation of a low Nickel diet lacked a control group. If confirmed, however, a patch test for metals in diabetes and nutrition centres might be suggested.

## Limitations

A potential limitation is that a low Nickel diet is relatively fiber poor and can lead to constipation in some patients, and we cannot give any information regarding the long-term consequences of consuming this special diet. Other limitations are the low sample size and the lack of a control group. Large cohorts of patients from multiple institutions and multivariate analysis are needed and tests should be carried out between different ethnicities, since this clinical study was performed only in the Italian population.

In this study, we could not evaluate males because of their small numbers and our findings are applicable only to females. Females were overrepresented in our study group, probably because more women are obese than men and Nickel allergy is more frequent in women than man. A larger sample would permit a sufficient number of male patients to be represented. Strategies to enhance the adherence to a low nickel diet program should also be adopted.

## Conclusion

This pilot observational analysis showed a substantially higher prevalence of Nickel allergy among overweight females, especially those with metabolic syndrome and fatty liver disease. A normocaloric low Nickel diet was effective in reducing BMI in this population. Further research is strongly needed to confirm these preliminary findings.

## Transparency Declaration

We affirm that this manuscript is an honest, accurate and transparent account of the study being reported; that no important aspects of the study has been omitted.
